# X-ray Tomography Coupled with Finite Elements, A Fast Method to Design Aerogel Composites and Prove Their Superinsulation Experimentally

**DOI:** 10.3390/gels8110732

**Published:** 2022-11-10

**Authors:** Genevieve Foray, Jaona Harifidy Randrianalisoa, Jerome Adrien, Eric Maire

**Affiliations:** 1 Université de Lyon, INSA-Lyon, Université Claude Bernard Lyon 1, CNRS, MATEIS, UMR-5510, 69621 Villeurbanne, France; 2 Institut de Thermique, Mécanique et Matériaux (ITheMM), Université de Reims Champagne-Ardenne, Campus Moulin de la Housse, CEDEX 2, 51687 Reims, France

**Keywords:** aerogel, silica, efficiency, tomography, homogenization, finite element, thermal, mechanical, simulation, measurements

## Abstract

Composite aerogels can include fibers, opacifiers and binders but are rarely designed and optimized to achieve the best thermal/mechanical efficiency. This paper proposes a three-dimensional X-ray tomography-based method for designing composites. Two types of models are considered: classical and inexpensive homogenization models and more refined finite element models. XrFE is based on the material’s real three-dimensional microstructure and/or its twin numerical microstructure, and calculates the effective conductivity of the material. First, the three-dimensional sample is meshed and labeled. Then, a finite element method is used to calculate the heat flow in the samples. The entire three-dimensional microstructure of a real or fictitious sample is thus associated with a heat flow and an effective conductivity. Parametric studies were performed to understand the relationship between microstructure and thermal efficiency. They highlighted how quickly a low volume fraction addition can improve or ruin thermal conductivity. A reduced set of three formulations was developed and fully characterized. The mechanical behavior was higher than 50 KPa, with thermal efficiencies ranging from 14 to $15\;\mathrm{mW}\cdot m\cdot K^{-1}$.

## 1. Introduction

Societal needs confronted by climate change together with government incentives now require reductions in energy consumption. Regardless of the application —buildings, industrial machinery or plants, or transportation—sustainable and cost-effective composites should meet the requirements of climate change. Due to their very high number of nanopores, silica aerogels are characterized by one of the lowest conductivities. They are therefore very interesting candidates for superinsulation. Aerogel-based materials with thermal conductivity ($\lambda_{}$) below $15\;\mathrm{mW}\cdot m\cdot K^{-1}$ belong to the group of materials called SIM (Super Insulation Materials). SIMs are the subject of unprecedented efforts in research and include vacuum panels and aerogel composites. Aerogel composites can be granular or super-insulating particles (SAP) glued or agglomerated by a binder and additives.

Many works have been dedicated to heat transfer in aerogel materials [1,2,3,4]. Heat transfer within these materials has two major routes, (through the gas phase in the pores and through the solid phase). Despite the fact that aerogels already have many industrial applications [3,5,6,7], their breakthrough in the building insulation market [8,9,10] is still expected. This specific application requires the mass production of a low-cost product with good mechanical properties and excellent thermal properties. However, contradictions may exist regarding the properties expected from the material. Also, experimental design is feasible but time consuming. Numerical design could help to open up new formulation routes once the numerical procedure has been validated.

Formulation trends to optimize these SIMs available in the literature comprise: (i) adding reinforcing fibers within aerogel slurry [11]; (ii) adding translucent polymer sheets on each side of the SIM; (iii) adding a binder [12]; (iv) and adding an opacifier [13,14]. Since the best additive materials have conductivities 10 (organic) to 40 times (mineral) higher than aerogel, they should be used in very low volume fraction, otherwise conductivity is impaired.

Three mechanisms are involved in composite heat transfer: conduction in the solid phase, conduction in the gas phase (air standing in pores), and thermal radiation. Heat transfer via the solid backbone depends on the skeleton structure and the skeleton morphology. Another thing to be taken into account is how the primary silica particles are connected, and how the Super-insulating Aerogel Particles (SAP)—either large, medium or small—, are fixed in three dimensions. Last but not least, the chemical core composition and the chemical surface composition of the hydrophobized silica can impair thermal efficiency. For a given temperature gradient through a SIM composite, heat is transferred by diffusing phonons via this complex chain of silica. Adding a low volume content of additional materials (binder, fiber, or opacifier) all along this backbone can disrupt the intrinsic low conduction heat transfer.

Heat transfer via the gas phase depends on the complementary phase of the backbone: the pore network. This network is bimodal. First, the pore size distribution within the aerogel network is well below seventy nanometers, which constitutes a gas confining environment and thus leads to extremely low gas heat transfer. Second, there are still gaps and spaces between the SAP and the additives which can be as large as a few microns. These induce significant gaseous heat transfer for a moderate volume fraction.

Finally, we must consider thermal radiation. The contribution of thermal radiation to the overall conductivity at room temperature is quite small in value (10% of total flux) but must be taken into account for optimization [2,14]. These small contributions are due to the highly scattered and absorbent nature of aerogel, as characterized by Fourier transform infrared spectrum measurements [13].

Future improvements in aerogel-based SIMs must consider the combination of the three mechanisms described. To date, the radiative mechanism is the most documented. Mie conditions (i.e., particle size < one third of the target radiation) are applied. Infrared opacifiers, mineral particles of a size of two to nine microns (e.g., carbon black or titanium dioxide) offer a cost-effective route to eliminate radiation. However, ensuring effectiveness requires a homogeneous dispersion of the three-dimensional opacifiers in the composite without the formation of agglomerates [14,15]. Thus, our computations and simulations will be focused on the two other primary mechanisms that are less documented.

In [16], Hrubesh et al. established a conductive and radiative model that assesses the contributions of each aerogel phase (pore/backbone), by taking as input data global values such as mean pore size, density, skeleton conductivity, refractive index, and extinction coefficient. They compared experimental and numerical thermal conductivities, versus densities. For low densities, the model failed as it underestimated gaseous conductivity. However, the model confirmed that the optimization path should first focus on decreasing the pore size, regardless of the material. Spagnol focused on how to simulate the contact point between aerogel particles, using a two-step numerical model [17], while other teams tried using hollow cells instead but encountered the same tendency to underestimate [18]. 

In addition, several homogenization models have been implemented to link the effective thermal conductivity to the global conductivities of dry air ($\lambda_{\mathrm{air}}$) and aerogel $(\lambda_{\mathrm{aerogel}}$), as well as to material characteristics such as Intergranular Porosity $(IP=1-Vf_{\mathrm{aerogel}}$) with $Vf_{\mathrm{aerogel}}$ equals the volume fraction of aerogel within pile up, and aerogel morphologies. They include the Serial model ($\lambda_{\mathrm{serial}}$) (Equation (1)), the Parallel model ($\lambda_{\mathrm{parallel}}$) (Equation (2)), the Hashin Strinkman model and the Maxwell Garnett model ($\lambda_{\mathrm{MG}}$) [19,20] (Equation (3)), which are well known and easy to handle. The Bruggeman model [21] offers an efficient way to include pore geometry based on X-ray tomography. Applied to glass particles and aluminum foam without nanoscale roughness [22], this model is effective and interesting for vacuum super-insulation products.

(1) $$\lambda_{\mathrm{serial}}=\frac{1}{\frac{\left(1-Vf_{\mathrm{aerogel}}\right)}{\lambda_{\mathrm{air}}}+\frac{Vf_{\mathrm{aerogel}}}{\lambda_{\mathrm{aerogel}}}}$$

(2) $$\lambda_{\mathrm{parallel}}=\left(1-Vf_{\mathrm{aerogel}}\right)\lambda_{\mathrm{air}}+Vf_{\mathrm{aerogel}}\lambda_{\mathrm{aerogel}}$$

(3) $$\lambda_{\mathrm{MG}}=\lambda_{\mathrm{aerogel}}\frac{2\;Vf_{\mathrm{aerogel}}\;\lambda_{\mathrm{aerogel}}+\left[1+2\left(1-Vf_{\mathrm{aerogel}}\right)\right]\lambda_{\mathrm{air}}}{\left[2-\left(1-Vf_{\mathrm{aerogel}}\right)\right]\;\lambda_{\mathrm{aerogel}}+Vf_{\mathrm{aerogel}}\;\lambda_{\mathrm{air}}}$$

Only a few patented aerogel formulations have claimed conductivities in the range of 18 to $35\;\mathrm{mW}\cdot m\cdot K^{-1}$ [23,24]; many other formulations include aerogel but have aimed at conductivity values from 35 to $70\;\mathrm{mW}\cdot m\cdot K^{-1}$ [25,26].

In practice, granular aerogels can be substituted for mineral fillers, as shown by formulation studies on concrete [27,28,29], plaster [30,31], and paper honeycomb [32]. Weigold and co-authors [33] provided some early notions on aerogel synthesis, while Li and co-authors [34] tailored aerogel chemical properties. This opened avenues for adding a small amount of aerogels to conventional insulating composites such as polyurethane board [35,36] and fiber composites [15,37,38,39], thereby boosting their thermal properties. Recent experimental work has focused on industrial demands such as three-dimensional aerosol printing [3] and designing superinsulation for transportation [40]. Three-dimensional X-ray tomography has been used to understand the occurrence of shrinkage cracks during synthesis in fiber aerogel mats [37,41] and during the service life of aerogel boards [11]. Aerogel grain brittleness and stack rebound while compacting aerogel granules were imaged and quantified [42]. To our knowledge, no study of the numerical optimization of SAP stacking and SAP pile-up has ever been published, although it could help to develop paths for formulating composites. The reason for this lack of studies is the very complex nanometric microstructure involved, and the lack of available characterization tools.

In this paper we investigate the numerical and experimental thermal properties of an aerogel-based Super Insulating Material (SIM). These are highly porous granular materials with nanorough contacts. Super-insulating Aerogel Particles (SAP) are mixed with a binder, then cast, and after drying form a SIM. Two types of SIM are studied: (i) no additives, SAP packing defined by its compacity and (ii) with additives, (SAP packing plus a binder). The voids in between the SAP, here called Intergranular Porosity (IP), and the binder volume fraction are global scale characteristics. An innovative technique based on X-ray microtomography image processing and finite element modeling is proposed to optimize the SIM formulation. The true three-dimensional morphology of the material is used to calculate the thermal conductivity, rather than global values. The technique, the model validation and the parametric study are presented in this paper. The best guesses given by simulation tools are then formulated and the real material fully characterized.

## 2. Results and Discussion

First the statistical parameters obtained with 3D tomography volumes are discussed. Then, these results are used as inputs for homogenization tools and the consequences on conductivity simulations ($\lambda_{\mathrm{MG}}$) are analyzed. Finally, XrFE model simulation ($\lambda_{\mathrm{XrFE}}$ effective thermal conductivity) results are shown and compared with real elaboration and thermal measurements ($\lambda_{\mathrm{mes}}$).

### 2.1. Statistical Results Gained on Porosity and Size Distribution

**Figure 1:**
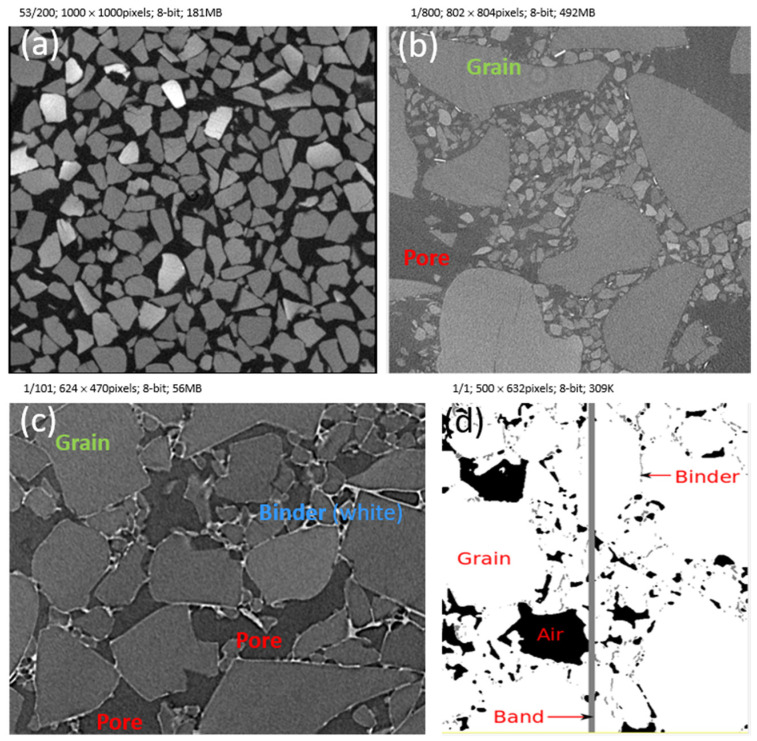
Tomography cross-section of aerogel composite. (**a**) Monomodal pile-up medium size grain IP = 36%; (**b**) bimodal aerogel pile up IP = 42%; (**c**) organic binder and bimodal pile-up IP = 19%; (**d**) defect simulation, binder band perpendicular to the heat flux.

**Figure A2:**
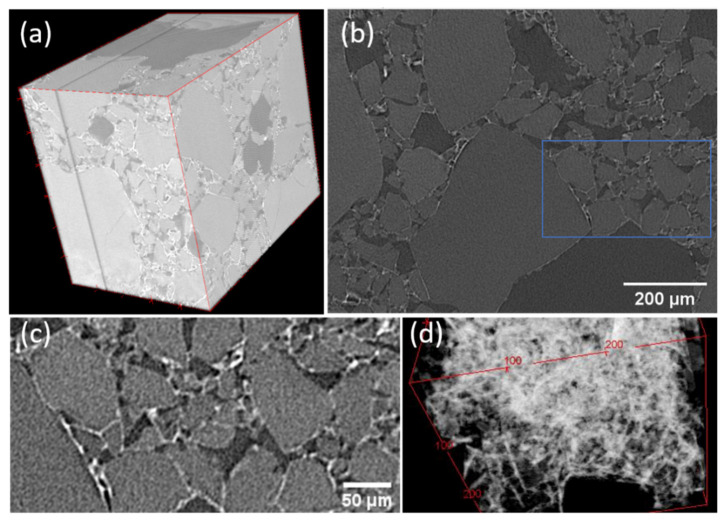
Composite aerogel with binder, and 3D-reconstructed ESRF X-ray tomogram to illustrate the post-treatments performed; (**a**) raw 3D volumes showing the three phases, (the binder, SAP, and IP); (**b**) cross-section resulting from (**a**) showing in white the binder has a discrete element in IP (outside SAP); (**c**) zoom in (**b**) (shown as a blue perimeter) showing that the organic binder either wraps SAP or connects SAP with fibrils; (**d**) after segmentation to separate the binder phase, (3D crop to show the connectivity of the binder phase).

Here, the main parameters studied to optimize the composite efficiency are the size distribution of the SAP (Figure 1a), the combination rate of small or large particles to optimize the porosities between the aerogel particles (Figure 1b), the nature of the contacts (Appendix A Figure A2, Figure 1c), the type of binder (Figure 1c), the process defects (Figure 1d) and the addition of opacifier.

The description of results always begins with pile up without addition. These are specific two-phase materials whose compacity equals the aerogel volume fraction, IP equals the pores between SAP, and their sum equals one.

#### 2.1.1. Compacity and Inter-Aerogel Particles Porosity (IP)

Mild compaction of the sample resulted in a 0.04-point increase in the particle volume fraction of the super-insulating aerogel, and thus improved thermal efficiency. This result confirmed a previous study [42] showing that harsh 50% strain compaction reduces the IP to 0.02. Optimizing the grain size distribution, and combining large and small particles, resulted in a 0.12-point decrease in porosity (IP), and thus much lower thermal conductivities.

By adding a binder, the IP is reduced by at least 50% and is less than 19%. This is quite unusual, and we assume it is due to volume shrinkage. The prismatic specimens are demolded just after wet forming, the specimen dries and its dimensions evolve. The volume shrinkage measurements on drying are 0.05 and 0.09 for the T and X binders, respectively. Previous studies reached those densities with aerogel granules using a permanent uniaxial pressure [42,43], and proved their thermal efficiency.

Adding opacifiers to the composite drastically reduced the volume fraction of pores measured with X-ray tomography. This formulation was expected to reach the lowest thermal conductivity.

Table 1 presents the density measured ρ on the materials and the tomography characteristics, including the IP, the volume fraction of Superinsulating Aerogel Particles (SAP) $Vf_{\mathrm{aerogel}}$, $Vf_{\mathrm{air}}$ the volume fraction of air, and $Vf_{b}$ and $Vf_{c}$ the volume fractions of specific phases. These are either the binder or the contacts between particles. The intergranular porosity (IP) calculated ranged from 6% to 48.3% among the formulations characterized. The volume fraction of aerogel measured was between 52.7 and 86%.

#### 2.1.2. Pore Size Distributions

Inside the aerogel particles, the pores were close to 10 nm in size and their three-dimensional connectivity was known [44]. Between these aerogel particles (named SAP), some pores were defined and their overall volume content was equal to Intergranular Porosity, the IP. Their pore size distribution also governed SIM efficiency. The smaller the IP value and size, the lower the thermal conductivity and the better the efficiency.

**Figure 2:**
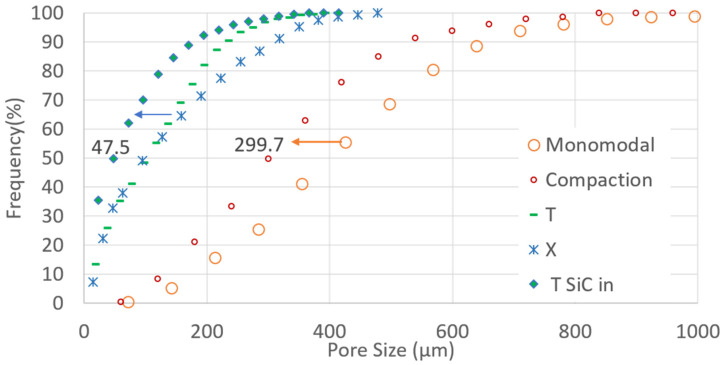
Pore size distribution determined by X-ray tomography, compaction, combining grain size, binding, and binding with SiC addition contribute to decreasing pore size.

Figure 2 shows that most of the pores become 100 µm smaller in size after a compaction process. The large orange dotted curve shifts to the left. Mixing small and large super-insulating aerogel particles (SAP) increases compacity (0.580 to 0.620) due to filling: up to hundreds of small particles replace air voids between the large particles. In addition, the values in Table 2 show that due to particle brittleness some large SAP (650 µm) fracture and give way to medium size SAP (420 µm), which also contributes to increasing compacity: Less spectacular, but noteworthy, is that the addition of binder removes pores sizes greater than 500 µm. Finally, the addition of opacifier particles further reduces pore $d_{50}$ values to 47.5 µm (Figure 2), and improves the overall pore distribution.

#### 2.1.3. Binder Volume Fraction

Since the binder is the weak link in formulations due to its intrinsic conductivity, it is necessary to optimize the quantity, localization, thickness and the thermal conductivity of the binder by performing simulations. Due to wet processing, some gradients may occur perpendicularly to the heat flow and the drying direction.

**Figure 3:**
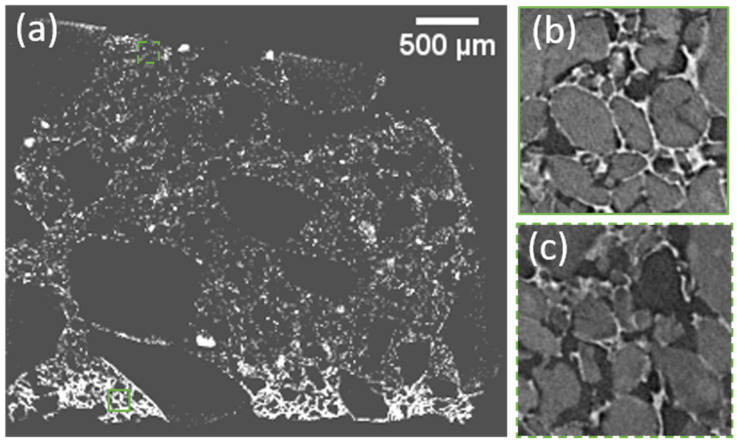
(**a**) Full cross-section of a segmented prismatic sample X binder, (pores and SAP are black), showing that on the bottom the volume fraction of binder is higher and the thickness of the binder is greater,(green squares locate zoom shown in (**b**,**c**)); (**b**) 200 µm × 200 µm bottom zoom (green square in (**a**)) showing SAP sealed by a thick continuous binder skin near the sample edge; (**c**) 200 µm × 200 µm top zoom (green interrupted line square in (**a**)) showing a thin and disrupted binder skin of a few microns thick, far from the sample edge. (**b**,**c**) the binder is white, SAP is light gray, and the IP is dark.

A full cross-section of the bar shape sample, as shown in Figure 3a, confirms that there is more binder at the bottom than at the top. $Vf_{b}$ equals 0.014 within the first 600-micron depth and then decreases to 0.006 at the top of the sample (Figure 3a). In both locations, the binder sealed particles and linked them, but this wall is continuous and reached 30 µm thickness at the bottom (Figure 3b), while it was disrupted and only a few microns thick at the top (Figure 3c).

This give a hint of the lowest thickness of binder that should be simulated and also a range of values for the binder volume content.

### 2.2. Homogenization Computed Thermal Conductivities with Tomography Volume Fraction as Input

**Figure 4:**
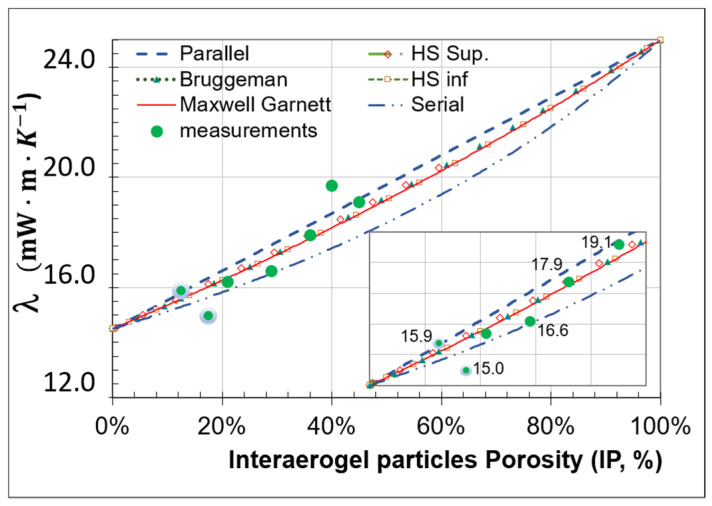
Homogenization simulated thermal conductivity values compared to experimental measurements (issued from [45]) as a function of inter-aerogel particle porosity (IP determined with X-ray tomograms). An insert zooms on data within the superinsulation range. Input values: $\;\lambda_{SAP}^{*}=$ $14\;\mathrm{mW}\cdot m\cdot K^{-1},\;\lambda_{air}^{*}=$ $25\;\mathrm{mW}\cdot m\cdot K^{-1}$.

Figure 4 shows how the thermal conductivity evolves and increases when the volume fraction of large pores increases (IP), and thus the volume fraction of SAP decreases. The lines of Parallel and Serial models define a large banana like area $1\;\mathrm{mW}\cdot m\cdot K^{-1}$ wide at 50% porosity. The Hashin-Strinkman model, the Bruggeman model, and the Maxwel Garnett model ($\lambda_{MG}$), are almost superposed in the super-insulation domain, as shown in the insert. 

All the model results justify the search for compactness, 20% fewer pores induce a $\Delta \lambda /\lambda_{\max Gar}=\frac{1.9}{15.4}=12\%$ more efficient composite.

The data of five out of seven experiments [45] are fairly approximated. For low porosity (IP = 14%) the models overestimate conductivity $\left(\Delta \lambda /\lambda_{mes}=\frac{0.9}{15}=6\%\right)$, for high porosity (IP = 40%) the model underestimates conductivity $\left(\Delta \lambda /\lambda_{mes}=\frac{-1.5}{19.7}=-8\%\right)$. The non-homogenous size of the pores within pile-up, the contact between the SAP, and the binder induced modifications may explain such differences.

By combining several runs of the homogenization model, we can compute a homogenized conductivity for the aerogel and the binder and then a homogenized conductivity for the pores and (aerogel + binder). This provides feasibility values starting with a fair compacity skeleton, for instance IP = 17.4%. For 0.6, 1.3 and 2% volume fractions of organic binder, Maxwell Garnett simulations give thermal conductivities equal to 16.3, 17.1, and $17.9\;\mathrm{mW}\cdot m\cdot K^{-1}$, respectively. These values overestimate the experimental values (green and grey dots of Figure 4 equal to $15\;\mathrm{mW}\cdot m\cdot K^{-1}$) but confirm that the addition of the binder should be as low as possible. Using an additional 1% volume fraction of the binder than the compulsory quantity to shape a panel sample causes a $\Delta \lambda /\lambda_{\max Gar}=\frac{1.3}{17.1}=8\%$ increase in the thermal conductivity.

When performing an inverse analysis, the intrinsic conductivity of SAP should be equal to $\lambda_{SAP}^{*}=$ $12\;\mathrm{mW}\cdot m\cdot K^{-1}$ to retrieve the experimental values. Shrinkage occurring as the SIM sample dries may cause a slight isostatic compression of each aerogel particle, thus the largest pore within the particles may collapse and the intrinsic conductivity values decrease.

### 2.3. Image Analysis and Computation Procedure with XrFE Code for Effective Themal Conductivity

**Figure 5:**
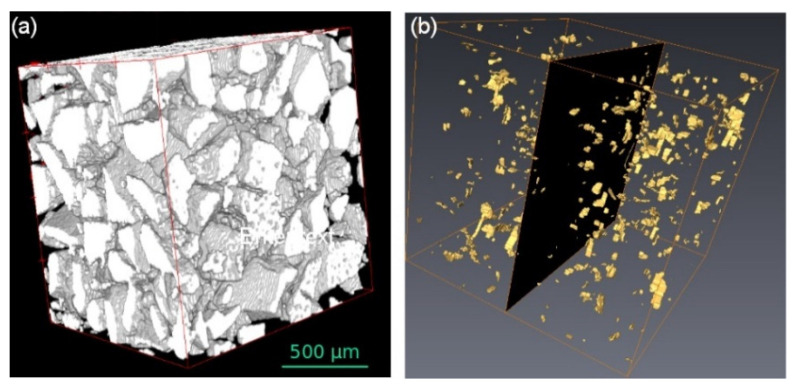
3D reconstructed X-ray tomogram illustrating the post-treatments performed: (**a**) segmentation and medium filter applied, with view of SAP only; (**b**) watershed applied, contacts between grains only viewed. The dark 2D square in the image illustrates a possible cross-section. (reprinted with permission from [46]).

**Figure 6:**
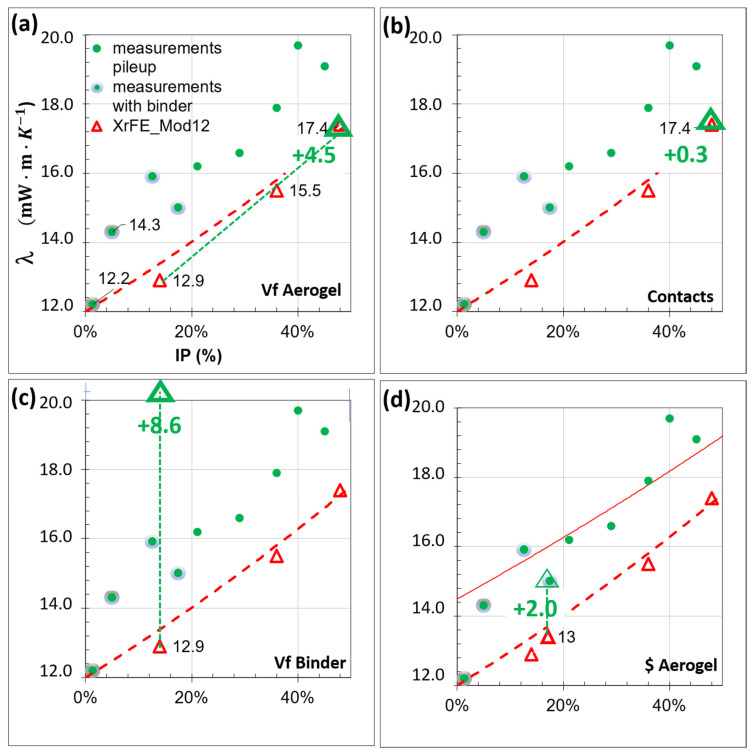
Parametric study on an aerogel composite with the XrFE model. (**a**) $Vf_{}$ of aerogel, (increasing compacity causes a sharp decrease in conductivity); (**b**) the thermal conductivity of the contacts,(including contacts in mesh, is neutral); (**c**) oganic binder, (filling pores with binder causes a huge increase in conductivity); (**d**) aerogel price and efficiency, (when using lower-priced, lower thermal intrinsic conductivity aerogel, XrFE and experimental conductivity are equal).

With XrFE, the size effects, the three-dimensional connectivity (Figure 5), and the non-homogenous material texture are fully implemented in the effective thermal conductivity simulation $\lambda_{XrFE}$ (see Equations (4)–(6) in Section 4). Thus, we can perform a sensitivity analysis. The input parameters and the simulation are summarized in Table 3, Table 4 and Table 5, while Figure 6 shows an overview of the sensitivity analysis. The experimental values and the Maxwell Garnet simulations (with a $\lambda_{SAP}^{*}=$ $14\;\mathrm{mW}\cdot m\cdot K^{-1}$) are shown as a reminder in Figure 6. 

#### 2.3.1. Aerogel Particle Size, Monomodal or Bimodal

The first set of simulations compares large and small SAP pileup. Monomodal lines in Table 3 show that 10% fewer pores and smaller pore size induce a $\Delta \lambda /\lambda_{XrFE}=\frac{1.6}{15.5}=10\%$ more efficient composite. XrFE was able to measure the reduction and its extent, so virtual tomography volumes could be used to tailor particle size distribution, thereby searching for efficient properties.

In the second set of simulations, the bimodal simulations are intended to find the optimal small/large particle ratio. The results in Table 3 show a moderate conductivity decrease down to $16.1\;\mathrm{mW}\cdot m\cdot K^{-1}$ while IP decreases by an amount of 0.04. 

Figure 6a shows at first glance the large efficiency gain (green dotted curve) due to higher compacity in the SAP, (and, therefore, lower IP values).

#### 2.3.2. Contacts

In a usual granular composite, contacts and interfaces are the keystone. Our model offers the unique opportunity to perform a sensitivity analysis for nanoporous materials such as SAP. Figure 5a shows a monomodal SAP composite, and Figure 5b shows only the contact domains in between SAP; the pores and particles are hidden. As long as the contacting material has low conductivity, (i.e. an organic binder or aerogel dust) the contacts do not hinder the material’s performance (Table 3). Filling all the contacts with a high conductivity material (i.e., a mineral binder) induces the smallest simulated increase $\Delta \lambda /\lambda_{XrFE}=\frac{0.3}{17.4}=1.7\%$. Figure 6b highlights that the contacts (0.3 efficiency loss) have a second-order parameter in SAP composite thermal efficiency optimization. A simulation with an optimized cellular material would provide a quite different view. Within the cellular microstructure, disrupting contacts and lowering their intrinsic conductivity both have a moderate impact on conductivity values.

#### 2.3.3. Binder

The binder phase is mandatory to transform the SAP into a panel composite. Starting with a fairly optimized skeleton with an IP equal to 14%, we first determined with XrFE simulation tools the conductivity of the upper bound (Table 4). How high are the organic and mineral composite conductivities if the IP is fully filled with a binder? The organic conductivity shows a rate of increase of 1.6 while the mineral shows a rate of increase of 3.5 (Figure 6c, green dotted curve). The organic SAP remains beyond the threshold that determines super-insulation, while the mineral SAP ($45.3\;\mathrm{mW}\cdot m\cdot K^{-1}$) is among the best mineral insulation materials.

When designing a composite, the consequences of usual processing defects must also be estimated. Let us simulate the worst case for thermal efficiency, a vertical binder band with no aerogel at all (+1%$Vf_{binder}$), standing parallel to a heat flux (Figure 1d). Table 4 indicates that a composite with such a defect has $17.2\;\mathrm{mW}\cdot m\cdot K^{-1}$ thermal conductivity, and thus possesses a considerable $3.4\;\mathrm{mW}\cdot m\cdot K^{-1}$ increase. The simulation also confirmed that binder bands perpendicular to the heat flux are not detrimental. Taking another view, a continuous 90-micron thick band of aerogel perpendicular to the flux induces a slight $-0.5\;\mathrm{mW}\cdot m\cdot K^{-1}$ thermal efficiency improvement. Increasing the processing rates on the aerogel fiber mats was also shown to be detrimental, as conductivity was multiplied by 1.5 and reached $21.5\;\mathrm{mW}\cdot m\cdot K^{-1}$ due to a large crack across the composite [47]. Homogenization models may estimate homogenous cracking but fail to simulate oriented cracks fully simulated with XrFE.

A large number of thermal simulations of IP/binder combinations reached the efficiency threshold (Table 4). IP and binder volume fraction are highly correlated parameters. the global value is important, but the microstructure also plays a major role. A tenuous binder content with a rather large IP provides the best SAP composite with a thermal conductivity equal to $13.2\;\mathrm{mW}\cdot m\cdot K^{-1}$ according to the simulation. 

While avoiding energy leaks, the SAP composite must be affordable meaning that it can be used widely. Figure 6d shows that switching to a cheaper aerogel price with intrinsic conductivity values equals $14\;\mathrm{mW}\cdot m\cdot K^{-1}$, so the SAP composite combines efficiency and price.

Figure 6 groups the results of the parametric analysis simulation with XrFE together with the plots of the homogenization models and the measurements performed.

### 2.4. SAP Composite Formulated and Characterized

**Figure 7:**
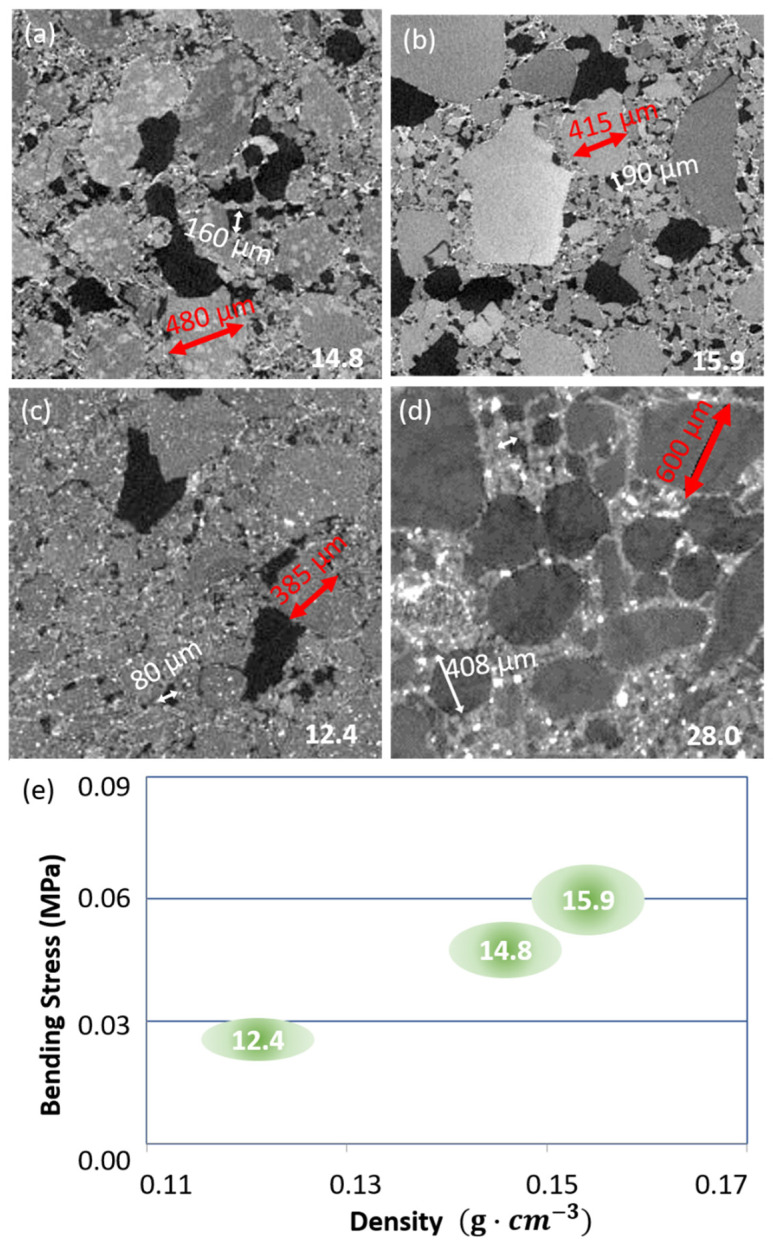
Tomography cross-section of aerogel composite. (**a**) SBA T composite $Vf_{binder}$ 2% IP = 17%; (**b**) SBA X $Vf_{binder}$ 2.0% IP = 12%; (**c**) SBA X SiC $Vf_{binder}$ 2% IP = 6%, please not the micron size isolated SiC particles paving space in white; (**d**) hydraulic binder, no quantitative analysis; (**e**) experimental values (flexural stress versus density and thermal conductivity measured given on graph in ($\mathrm{mW}\cdot m\cdot K^{-1}$) issued from ref. [45,48]).

The thermal conductivities of the commercial formulation with the hydraulic binder and the organic formulation are within the range predicted by the XrFE model. The parametric study sheds light on the role of the binder in ensuring low conductivity, so several trials were performed: organic binder with a small and large size surfactant, SiC addition, and hydraulic binder. Figure 7 shows the microstructures after elaboration and the material inside Figure 6d). All the organic and mineral composites show a bimodal porosity (Figure 7).

The pore shape is rounded and smooth for the mineral binder (Figure 7d), whereas it is angular and rough for the organic binder (Figure 7a–c). The formulation by semi-dry casting or by projection, modifies the microstructure although the raw material SAP is identical.

The IP is halved when SiC additions are used (Figure 7a–c). The binder at the local scale decorates the SAP with a leopard skin pattern in Figure 7a, it wraps the SAP in Figure 7b, and it forms a cement between the pores and large grain in Figure 7d.

Particles with high attenuation are visible in Figure 7c. These are the SiC particles and their distribution is homogenous all over the volume. Their size shows that SiC particles did not agglomerate during elaboration. This ensures efficiency because the radiative conductivity requires a high Z number and an adequate micron size to be reduced. The hydraulic binder composite also shows particles with high attenuation, but most are larger in size (a few tens of microns) and form agglomerates.

SAP have distributed sizes in all formulations. Those associated with the organic binder are polyhedral, with sharp angles; those used with the hydraulic binder have a smoother, rounded shape.

**Figure 8:**
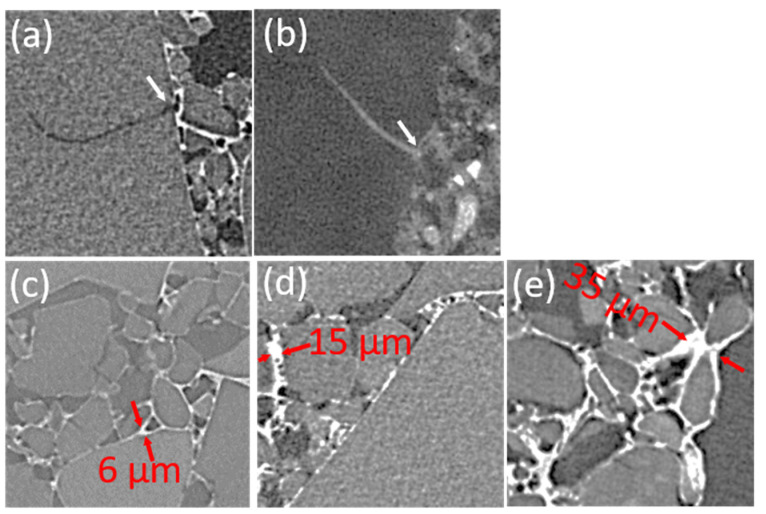
200 × 200 cross-section in the X-ray tomogram, microstructure details. (**a**) organic binder wrapping aerogel particles without binder intrusion inside aerogel particle cracks and fracture lines; (**b**) mineral binder filling space between aerogel particles, and also filling aerogel particle cracks and fracture lines; (**c**) organic binder T, ESRF, low thickness binder skin, and adhesive contact between particles; (**d**) organic binder X,— few medium thickness binder skins, including aligned air bubbles. (**e**) organic binder X,— numerous large thickness binder skins and regular thickness skins showing disrupted adhesion to particles and large air gaps in between. White arrows show crack openings, and red double arrows measure binder thickness perpendicular to aerogel particles.

Tomography volumes also confirmed that organic binder remains around SAP particles and never fills the pores or the open cracks that can occur within SAP (Figure 8a). The hydraulic binder behaves differently and can fill the cracks (Figure 8b). The lowest conductivity measured is associated with the microstructure in Figure 8c, with a discontinuous binder skin and a few connection points six microns in size. In Figure 8c, the intermediate conductivity is associated with continuous binder skin. Some binder strips are 15 microns thick and include small pores. Figure 8d, shows the thickest organic binder skin, decohesion between the binder and SAP, and a large binder plug in between SAP. 

## 3. Conclusions

This paper provided a detailed method that uses X-ray tomography to investigate aerogel based Super Insulating Material (SIM). Thus, the statistical data fully described pore size, particle size, IP (Inter superinsulating aerogel particles Porosity) and binder features. Indeed, the three-dimensional microstructure revealed all the interactions between aerogel, binder and mineral additives.

The detailed study on compactness showed the interest of combining bimodal particles to minimize inter-particle pores. Tomography confirmed the decrease of the median diameter during mixing. It was accentuated in the presence of the opacifiers which are hard grains and boosted the decrease of IP.

The revised homogenization models demonstrated beyond doubt the interest of densification in SAP but did not correctly describe the presence of a binder. Thermal conductivities were underestimated at large IP and overestimated at small IP. The conductivity of SAP composites including organic binder was overestimated, suggesting that the binder blocks thermal radiation, decreases SAP intrinsic conductivity or changes pore connectivity.

An innovative technique (XrFE) was proposed based on X-ray microtomography image processing and finite element modeling to optimize SIM. The tomography volumes were processed so as to create a meshed volume that incorporates the intrinsic conductivity of each phase (aerogel, binder, pores and contacts). True 3D material morphology was used to compute the thermal conductivity rather than global values. The input volumes either represented real materials that were characterized or numerical twins that were improved.

The XrFE numerical process was validated, formulation hints were correctly ranked, and the results proved that the model is sensitive. Abaqus Simulations shed light on two formulation parameters, -the inter aerogel particles porosity IP and the binder type and volume fraction. Simulations also confirmed that contacts between SAP are second-order parameters, and that defects parallel to heat flux are first order. 

The best guesses given by the simulation tools were formulated and fully characterized. Their conductivities met the requirement. The microstructure analysis revealed that the binder might be discontinuous around the SAP, decorating aerogel particles with a leopard skin pattern. They provided good thermal efficiency, although their IP was not the lowest. The best efficiency was achieved with the addition of an opacifier and a discontinuous binder skin. Some easier-to-process formulations wrapped SAP particles with a continuous binder skin. The weakest link of the formulation was between thick coated grains. The formulations with large conductivity values showed some SAP coating decohesion.

## 4. Materials and Methods

The materials studied are nanostructured silica aerogel granules obtained by a two-step sol-gel process and dried at ambient condition. In particular, two kinds of this material are analyzed: a granular aerogel pile-up and a granular aerogel composite (aerogel + binder system). The organic composite has the following composition in dry weight fraction: organic binder (0.11), large granules(0.53), and small granules(0.35), (please refer to [12] for additional information on the binder). The characteristics of the samples studied are summarized in Table 1 in growing order of complexity, (first the aerogel pile-up only, then the composite ones). Some formulations were characterized with several resolutions down to 0.54 µm, a single formulation is provided in Table 1. 

The 3D reconstructed volumes are obtained by high resolution X-ray microtomography. The general principle of this technique can be found in [49], its application to porous material at temperature in [50], and the way it helps understand aerogel composite mechanical behavior in [11]. Two different setups with complementary resolutions are used so as to obtain the tomogram required for computations: a standard laboratory tomograph (MATEIS, INSA-Lyon), and the line ID19 of a synchrotron radiation tomograph located at the European Synchrotron Radiation Facility (ESRF) in Grenoble (France). This enables pixel resolution down to 500nm of interest to describe the binder/particle interaction. The volumes studied are up to a few cubic centimeters (with a 1.4 µm resolution). 

The ESRF beam line offered attractive measurement possibilities due to a large sample/source distance (150 m), and a small X-ray source size (100 µm). The electron beam energy was fixed at 19 keV in order to produce hard X-rays; these X-rays are monochromated with a double-vertical silicon single crystal.

Concerning the MATEIS EasyTom Nano tomograph manufactured by the company RX Solutions, its X-ray source is operated with a LaB6 cathode at a voltage of 60 kV. The detector in this setup is a Hamamatsu CCD camera with a pixel size of 12 µm. 

The atomic number of Silicon is fourteen, which is a low atomic number value. As a result of this and of the aerogel porous structure, very little of the emitted X-rays are absorbed by these materials. It thus makes it hard to make the distinction between the air phase and the aerogel phase, as they have similar X-ray absorption properties. The microstructure observation by sensitive phase contrast results in the enhancement of the contrast between different chemical phases. 

Aerogels grains are piled up in a cylindrical container. The container has a thin paper side so that it will not influence the X-ray tomography. The diameter of the container is equal to ten times the median size of the aerogel particles. This value leads to a sample large enough to be significant, but not excessively X-ray absorbent. 

The composites with binders are solid materials. They are sculpted into approximatively match-shaped samples used for the tomography. The sides of the square section of the sample measure around 0.5–1 mm, depending on the required resolution. As the composite appears to be very brittle, it is quite difficult to have a square side dimension lower than 0.5 mm. As a result, high resolution tomographs with a voxel size smaller than 0.5-micron tend to be local tomography.

The X-ray stack of image treatment and volume meshing is detailed in Appendix A, while the thermal conductivity computation is explained thereafter. The work of Ulrich [51] showed very early that although geometrically attractive, cubic elements exploded the computation time and imposed VERs that were too small for property simulations. A previous study on the thermal conductivity of highly porous foam [52] was used here to define the optimal tetrahedral mesh density. This research on porous foam also proposes and describes the tools for dealing with the singularities of the material.

The total thermal conductivity can be expressed as the sum of conduction and radiation contributions. The contribution of conduction gives the effective thermal conductivity $\lambda_{\mathrm{eff}}$, expressed as

(4) $$\lambda_{\mathrm{eff}}=\lambda_{SAP}^{*}+\lambda_{air}^{*}+\lambda_{binder}^{*}$$

where $\lambda_{SAP}^{*}$, $\lambda_{air}^{*}$ and $\lambda_{binder}^{*}$ binder stand respectively for the thermal conductivity of the solid (the super-insulating aerogel particles), the gas (i.e., air in between the super-insulating aerogel particles) and the binder phase. The asterisks represent the properties of the porous materials to distinguish them from the properties of bulk substances. The thermal conductivity of the gas in cellular materials can be calculated with Equation (5) proposed by Glicksman [53] and used successfully in [54].

(5) $$\lambda_{air}^{*}=\left(1-\rho^{*}\right)\lambda_{air}$$

$\lambda_{air}^{*}$ is the bulk conductivity of the gas substance, $\rho^{*}$ is the relative density.

The gas conduction can be estimated by Equation (5). However, the calculation of the solid conduction is not an easy task. Strong numerical methods, such as the finite element method, are essential (for instance—those used for Voronoi’s foams [54]. 

In this work, tomography volume post-treatments provide a single mesh volume with the solid phase, the air, the binder, and the contacts. This is the input used to compute the aerogel composite’s effective thermal conductivity $\lambda_{\mathrm{XrFE}}$. By applying a thermal gradient to the sample face studied, Fourier’s law (Equation (6)) gives the heat flux through the sample.

(6) $$\lambda_{\mathrm{XrFE}}=\frac{L}{S}\frac{Q}{\left|\Delta T\right|}$$

where *L* is the sample thickness, *S* is the sample face surface, ΔT/L is the thermal gradient applied on the two opposite faces, and *Q* refers to the heat flux crossing the sample over the surface area *S* (*S* surface is normal to *L*).

The Finite Element Solver Abaqus, is used here to perform the computation of the main unknown *Q*: the nodal temperature and constant heat flux (within an element) will thus be obtained.

## Figures and Tables

**Table 1:** First SIM Formulations characterized by their type, and the volume reference and X-ray tomography features ($IP=1-Vf_{\mathrm{aerogel}}\;$ equals free space between SAP, $Vf_{\mathrm{air}}$, volume fraction of air in IP and specific phases quantified with their volume fractions: $Vf_{c}$ contacts and $Vf_{b}$ binder.

Aerogel Pile-up_Binder	Ref. Vol. ^(1)^	Resolution ^(2)^ (µm)	ρ $\mathrm{Density}\;(g\cdot {\mathrm{cm}}^{-3})$	IP	$Vf_{\mathrm{aerogel}}\;\mathrm{SAP}$	$Vf_{\mathrm{air}}$ Air	Specific Phase
Monomodal_No Binder	1	4.30	0.069 ± 0.005	0.483	0.517	0.483	No
	2	4.30	0.076 ± 0.005	0.360	0.640	0.360	No
Monomodal Compaction	L	13.5	0.09 ± 0.01	0.410	0.590	0.410	No
	LC	13.5	0.11 ± 0.01	0.370	0.630	0.370	No
Bimodal No Binder	4	3.00	0.069 ± 0.005	0.483	0.516	0.482	Vfc = 0.001
	3	4.30	0.076 ± 0.005	0.360	0.640	0.358	Vfc = 0.002
Bimodal_Organic_Binder	T	1.00	0.149 ± 0.005	0.190	0.790	0.190	Vfb = 0.020
	X	1.00	0.163 ± 0.005	0.120	0.860	0.120	Vfb = 0.020
Bimodal Organic Binder SiC	TSiC	1.00	0.155 ± 0.005	0.060		0.060	not meas.

^(1)^ 1 to 4 are volume number, L: aerogel particles without compaction, LC: L with compaction, T stands for a tiny surfactant within the organic binder, X stands for a large size surfactant, TSiC, stands for T binder system plus some opacifiers. ^(2)^ Resolution provides the pixel resolution used for X-ray tomography.

**Table 2:** Inter-Particle pores and Superinsulating Aerogel Particles, average sizes measured with a 3D grains growth procedure and X-ray tomography.

Volume Name and Features	Values Measured by Grain Growth within 3 D Tomography Volumes	
	$\mathrm{Particles}\;D_{50}{}^{\left(1\right)}\;(µm)$	$\mathrm{Pores}d_{50}{}^{\left(2\right)}\;(µm)$
Large SAP	650	380
Small SAP	30	22
Pileup with 60% Large SAP	$D_{L\_50}=420$	$d_{L\_50}=200$
+40% Small SAP	$D_{S\_50}=60$	$d_{S\_50}=50$

^(1)^ $D_{50}$, 3D average size of aerogel particles in a monomodal pileup, and average size of each population in a bimodal pile-up composite $D_{L\_50}$ for the Large one and $D_{S\_50}$ for the small one. ^(2)^ $d_{50}$, 3D average size of pores between aerogel particles in a monomodal pileup; $d_{L\_50}$, 3D average size of pores between larges particles in a bimodal pile-up composite; $d_{S\_50}$, 3D average size of pores between small particles in a bimodal pile-up composite.

**Table 3:** Pile-up compacity parameter study, inputs and XrFE simulated conductivities.

XrFE Volume Used as Input	Ref. Vol	$\mathrm{Input}\;\mathrm{Thermal}\;\mathrm{Conductivities}\;(mW\cdot m\cdot K^{-1})$		IP (%)	$\mathrm{XrFE}\;\mathrm{Simulated}\;\mathrm{Conductivities}\;(mW\cdot m\cdot K^{-1})$
	^(1)^	$\lambda_{SAP}^{*}{}^{\left(2\right)}$	$\lambda_{air}^{*}{}^{\left(3\right)}$	$Vf_{air}$	$\lambda_{XrFE}\;{}^{\left(4\right)}$
Monomodal Pile-up	L	12	25	0.48	17.1
	S			0.36	15.5
Bimodal Pile-up	L80	12	25	0.42	16.5
	L60			0.38	16.1

^(1)^ monomodal pile-up: L, with large SAP, S, with smal SAP, bimodal pile-up: L80 with 80% volume fraction of large SAP +20% of small SAP, L60, with 60% volume fraction of large SAP+ 40% of small SAP. ^(2)^ $\;\lambda_{SAP}^{*}$ intrinsic thermal conductivity of an aerogel superinsulant particle monolith. ^(3)^ $\;\lambda_{air}^{*}$ intrinsic thermal conductivity of static dry air. ^(4)^ $\;\lambda_{XrFE}$ effective thermal conductivity simulated with the tomography X-ray 3D volume and a coupled finite element model see details in Section 4 and Appendix A.5.

**Table 4:** Contact parameter study, inputs and XrFE simulated conductivities.

XrFE Volume Used as Input	$\mathrm{Input}\;\mathrm{Thermal}\;\mathrm{Conductivities}\;(mW\cdot m\cdot K^{-1})$			$\mathrm{XrFE}\;\mathrm{Simulated}\;\mathrm{Conductivities}\;(mW\cdot m\cdot K^{-1})$
	$\lambda_{SAP}^{*}$	$\lambda_{air}^{*}$	$\lambda_{contact}^{*}{}^{\left(1\right)}$	$\lambda_{XrFE}\;$
No contacts	12	25	0	17.4
Low contacts			12	17.4
High contacts			1000	17.7

^(1)^ $\;\lambda_{contact}^{*}$ intrinsic thermal conductivity of contact betwen Superinsulant Aerogel Particles.

**Table 5:** Binder and IP parameter study, inputs and XrFE simulated conductivities.

XrFE Volume Used as Input ^(1)^	$\mathrm{Input}\;\mathrm{Thermal}\;\mathrm{Conductivities}\;(mW\cdot m\cdot K^{-1})$			$Vf_{binder}(\%)$	IP Measured (%)	XrFE Thermal Conductivity $\left(mW\cdot m\cdot K^{-1}\right)$	
	$\lambda_{SAP}^{*}$	$\lambda_{air}^{*}$	$\lambda_{b}^{*}$			$\lambda_{\mathrm{XrFE}\_\mathrm{Organic}}\;{}^{\left(2\right)}$	$\lambda_{\mathrm{XrFE}\_\mathrm{mineral}}\;{}^{\left(3\right)}$
Upper Bound	12	25	200–1000	14.0	None	21.5	45.3
Lower Bound				None	14.0	12.9	12.9
Vertical defect band	14	25	200–1000	1.0 + 1.0	17.4	17.2	-
No defect band				1.3	17.4	13.8	15.1
Moderate	12	25	200–1000	1.3	14.0	13.6	14.8
Low				0.3	12.7	13.7	13.8
Tenuous				0.1	17.5	13.2	13.3

^(1)^ upper bound, all IP is filled with binder no air between SAP, vertical defect band a defect a few microns thick parallel to heat flux is inserted see Figure 1d, Moderate to tenuous, decreasing content of binder in composite formulations. ^(2)^ $\lambda_{XrFE\_Organic}$,organic effective thermal conductivity computed with XrFE model using polymers to bind aerogel pile- up. ^(3)^ $\lambda_{XrFE\_mineral}$, mineral effective thermal conductivity computed with XrFE model using lime plaster to bind aerogel pile-up.

**Figure A1:**
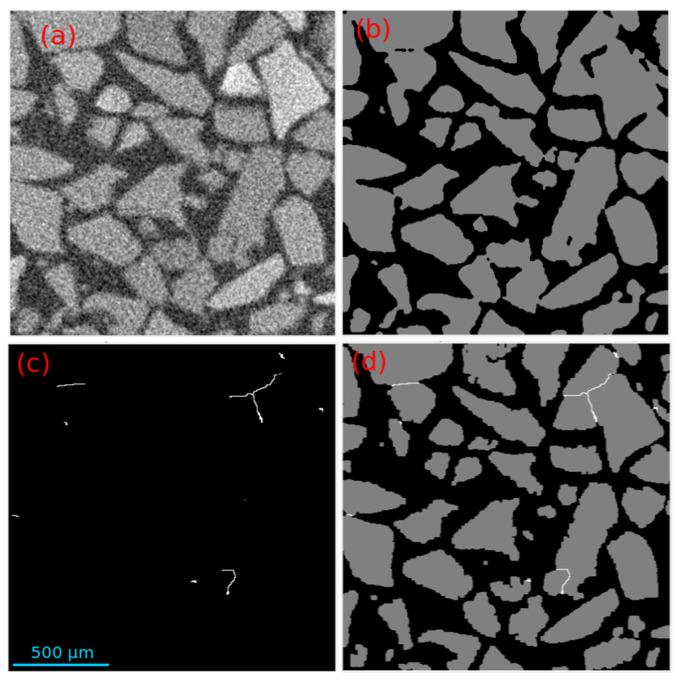
Cross-sections within the 3D reconstructed X-ray tomogram to illustrate the post-treatments performed: (**a**) raw monomodal SAP pileup; (**b**) segmentation and medium filter applied, two phases only viewed: air in pores and SAP; (**c**) watershed applied, contacts between SAP only viewed; (**d**) all phases (pores, SAP and contact between SAP (color code black, gray, white)).

**Figure A3:**
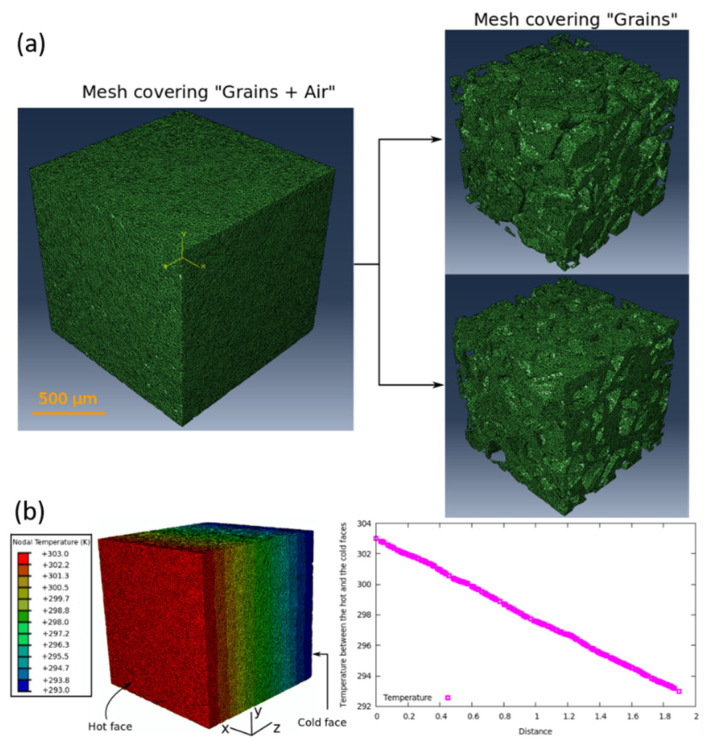
Meshed volumes based on 3D-reconstructed X-ray tomogram: (**a**) monomodal SAP composite recombined or not; (**b**) nodal temperature view within a bound bimodal composite.

## Data Availability

Not applicable.

## Appendix A

The refined tomography steps (experiments and postprocessing) enabled the microstructure description and numerical computation of the thermal efficiency We explain the underlying process in this appendix; the statistical results are presented in the article

Image analysis is straightforward whenever the observed phases within the material are highly contrasted and are fully separated. When poor contrast is encountered (low density or low X-ray attenuation), the shape factor (spherical particles, fibers) may help. Aerogel-based materials are therefore tricky and delicate, contacts are faceted, and their shapes and sizes are not uniform at all. Therefore, we provide below the segmentation with all the details.

### Appendix A.1. Segmentation to Separate Phases

We first consider SAP pile-up without a binder. In such samples, two phases are expected: the SAP and the surrounding air-filled pores. The surrounding air will be referred to as Intergranular Porosity (IP) in the following. Software Image J is used for phase segmentation. Let us consider a representative 3D reconstruction of an aerogel stack, whose sizes are 1600 × 1600 × 1600 µm^3^ and the voxel resolution 1 equals 4.3 µm. In this example, the SAP are monomodal, but this procedure is applicable to multimodal pileup as well. Even in an average quality 3D reconstructed volume (see a cross-section in Figure A1a), we can easily separate two phases: the black phase (corresponding to the X-ray absorption by the air) and the grey phase (corresponding to the X-ray absorption by the SAP). Some gray phases are brighter than others, meaning that there are at least two SAP populations, one denser than the other. This absorption gradient also occurs within some given Super-insulating Aerogel Particles, which also reveals possible internal density gradients.

Due to these gray level gradients, the segmentation of a phase on a 3D tomogram named thresholding required special care. Thresholding consists of converting a grayscale image into a binary image by defining a grayscale cutoff point. After this step, post-processing is performed on the binary images to remove noise using a “Median 3D” filter several times. This results in a clear binary section with two well identified phases (Figure A1b). The numerical color code chosen, associates gray with SP, and black with an IP full of air.

### Appendix A.2. Watershed Treatment to Retrieve Contacts

The thresholded cleaned volume is then processed using the Watershed module of the software Avizo to capture in 3D the contact surfaces between the SAP. The Watershed Split process divides clustered particles into separated labeled particles and thus allows for quantification and accurate geometric characterization. The contact planes are observed in 3D; the contacts correspond to interrupted white “lines” or white dots in 2D.

### Appendix A.3. Volumes to Ensure Quantitative Phase Analysis

Thus, from the initial volume describing a pile-up, four 3D reconstructions were built and labeled to perform the quantification. The first one includes only SAP (Figure A1a), the second one includes only intergranular porosity (IP), and the third one includes only contact zones (Figure A1b). Finally, these three volumes are recombined (Figure A1d), so as to produce a numerical twin microstructure simulating the real one. The twin volume identifies contact domains as a material phase. The numerical color code is enriched: the contact phase is white in the cross-section views and gold in the 3D models (report to Figure 5 main text).

These volumes then give access to true physical characteristics: the volume fraction of each phase in all directions, the overall volume fraction of interest for the homogenization tools, the size distribution, the geometry properties (Inertia, Shape factor), the connectivity and the tortuosity.

### Appendix A.4. Meshing Volume to Enable Thermal Simulation

The technique for obtaining voxels [55] is easy to implement, but it generates an extremely high number of nodes (8-node cubic elements). The technique adopted in this work first generates triangulated closed surfaces delimiting each phase [56]. Then, it meshes only these closed surfaces with 4-node tetrahedral elements. Thus, building the finite element mesh from our tomogram volumes is performed in three steps:

- Binarizing the grey level images to enhance the resolution of the boundaries;
- Geometrical definition of these phases by triangular facets;
- Tetrahedral volume meshing.

A 3D view of the volume meshes is available in Figure A3. Consider as an example, a medium grade SAP packed up to a 0.069 density. The mesh is composed of 356,062 nodes, and 2,035,521 tetrahedral elements with and average size of 25 µm. An algorithm developed at MATEIS then allows for the creation of subgroups in any volume with two or more phases. Here, each element has a label that indicates the contact nature, (for instance, aerogel/aerogel, aerogel/pore or pore/pore).

### Appendix A.5. Pile up with Binder, Post-Treatment Used for Aerogel Composites

By pushing the resolution of the tomograph apparatus (ESRF), an additional weakly connected phase of low thickness is clearly observable (Figure A2a): the binder. A close look at the volumes reveals that the binder is highly anisotropic. It occurs either in the IP (Figure A2b) as discretely connected branched fibrils (Figure A2c), or as a thin uneven film wrapping several grains (Figure A2d).

Here, we explain the process with the hydraulic binder formulation. When analyzing a large volume, three absorption levels are observed in increasing order: black represents the air in the IP, grey the aerogel grains and white the organic binder. Each phase has a specific gray level at this resolution (1 voxel = 1.4 µm), so the images are separated into three using the following gray level limits:

- 3D segmentation of the binder phase, showing a gray level close to 225, then binarization to create a reconstructed volume with only the binder;
- 3D segmentation of the air phase, (gray level near zero, manual adjustment of the upper boundary), resulting in a reconstructed volume of only pores.
- Combination by addition of the two previous volumes, IP and binder.
- This volume is then subtracted from the raw volume to recover in a volume the last phase of aerogel grains.
- Each of the 3 volumes (particles, air and binder) is meshed with the Avizo software.
- The 3 meshed volumes are recombined into a single volume and thermal or mechanical simulations are performed.

A qualitative view of the mesh generated for a bonded aerogel composite, including a small surfactant and compacted to a density of 0.127, (Volume 5, BPU_SBA_X) is not shown here. This mesh consists of 240,091 nodes and 1,351,729 tetrahedral elements; the average tetrahedron size is 4.5 µm. This mesh volume is used to calculate the heat flux with the sample as explained in Section 4. The effective thermal conductivity is then estimated for each sample studied.

The Figure A3 below shows the meshed volume of aerogel and several nodal temperature views during the simulation run.
